# Branched hybridization chain reaction—using highly dimensional DNA nanostructures for label-free, reagent-less, multiplexed molecular diagnostics

**DOI:** 10.1038/s41378-019-0076-z

**Published:** 2019-08-12

**Authors:** Gaolian Xu, Mingliang Lai, Rab Wilson, Andrew Glidle, Julien Reboud, Jonathan M. Cooper

**Affiliations:** Division of Biomedical Engineering, James Watt School of Engineering, University of Glasgow, Oakfield Avenue, Glasgow, G12 8LT UK

**Keywords:** Electrical and electronic engineering, Biosensors

## Abstract

The specific and multiplexed detection of DNA underpins many analytical methods, including the detection of microorganisms that are important in the medical, veterinary, and environmental sciences. To achieve such measurements generally requires enzyme-mediated amplification of the low concentrations of the target nucleic acid sequences present, together with the precise control of temperature, as well as the use of enzyme-compatible reagents. This inevitably leads to compromises between analytical performance and the complexity of the assay. The hybridization chain reaction (HCR) provides an attractive alternative, as a route to enzyme-free DNA amplification. To date, the linear nucleic acid products, produced during amplification, have not enabled the development of efficient multiplexing strategies, nor the use of label-free analysis. Here, we show that by designing new DNA nanoconstructs, we are able, for the first time, to increase the molecular dimensionality of HCR products, creating highly branched amplification products, which can be readily detected on label-free sensors. To show that this new, branching HCR system offers a route for enzyme-free, label-free DNA detection, we demonstrate the multiplexed detection of a target sequence (as the initiator) in whole blood. In the future, this technology will enable rapid point-of-care multiplexed clinical analysis or in-the-field environmental monitoring.

## DNA detection: enzyme-free amplification

A hybridization chain reaction permits multiplexed and label-free DNA detection. Nucleic acid detection generally requires a suitable enzyme to amplify the typically low concentration of target nucleic acid sequences, as well as careful control of temperature. However, this limits the ability for multiplexing strategies. Now, a team led by Jonathan M. Cooper at University of Glasgow in the UK report the use of DNA nanoconstructs to enhance the molecular dimensionality of hybridization chain reaction (HCR) products, enabling label-free multiplexed detection; the use of a 3-arm DNA construct creates additional sites to initiate a chain reaction with high efficiency, removing the need for otherwise complex labeling approaches.

## Introduction

Sensitive and specific nucleic acid detection has generally relied upon enzymatic amplification of samples to enhance assay performance, using established methods, including thermal cycling methods (e.g., polymerase chain reaction, PCR) or isothermal methods. These methods have enabled many important biological applications associated with analytical biotechnology, including high-throughput DNA analysis and highly sensitive DNA biosensors^1^. Difficulties in performing controlled thermal cycling for PCR, outside of a well-equipped laboratory, have led to the development of a range of isothermal methods, such as loop-mediated isothermal amplification, LAMP^2^, rolling circle amplification, RCA, strand displacement amplification, SDA^3^, cross-priming amplification, CPA^4^, and helicase-dependent amplification, HAD^5^. These latter assays are easier to implement (as they do not require precise control of temperature to enable thermal cycling), although their complexity, due to the numbers of primers required, has limited the extent to which multiplexed, multi-analyte isothermal assays can be implemented.

Both PCR and isothermal methods also involve enzyme-mediated amplification, which places additional constraints on the storage of reagents (e.g., to ensure enzyme stability) and sample preparation (to avoid enzyme-inhibiting species). To overcome these limitations, hybridization chain reactions have been developed as a DNA amplification system that is both isothermal and enzyme-free^6^. The mechanism generally involves two coexisting hairpin DNA monomers, which, upon introduction of an initiator, cascade through a series of hybridization reactions (based on the opening of the hairpin structure) to produce long DNA polymers^7^. The fact that the amplification is enzyme-free has prompted interest in both in vivo^8^ and in vitro assays, including its combination with other technologies, such as pyrene chemistry^9^ and electrochemiluminescence^10^. In common with other isothermal amplification reactions, however, the introduction of a multiplexing capability in HCR has been limited, because the linear growth of the final product in response to the initiator (or target sequence)^6^ requires a complete set of DNA initiators (in most cases, two hairpin DNA structures, at high concentration). This reaction condition has led to the high probability of cross talk (or interference) between the pathways by which different analytes are amplified. This subsequently has resulted in practical limitations in the implementation of HCR.

**Fig. 1:**
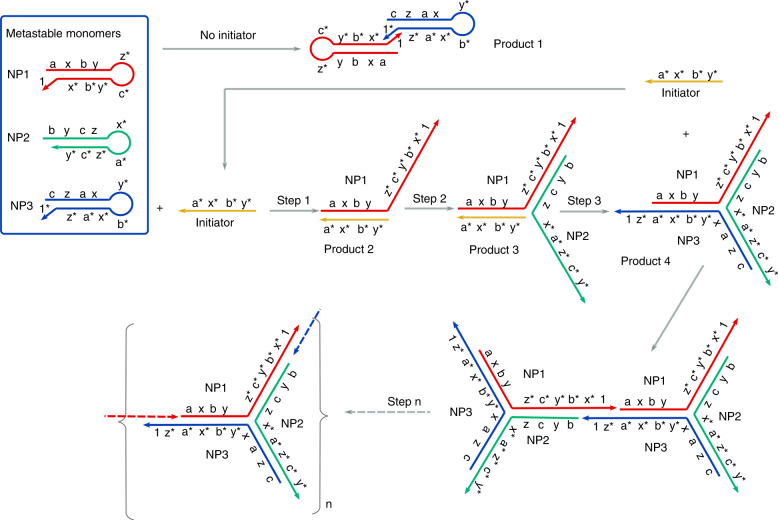
Mechanism of the branched HCR reaction. The basic linear implementation of HCR is modified by adding segments (1, a, b, c, x, y, and z, 1*, a*, b*, c*, x*, y*, and z*). The letter of the segment with a star is complement to the segment marked with the same letter. Each segment contains six nucleotides in length, while 1,1*, and 2 are 20 bp. The sizes of each product are provided in Table S1

Here, for the first time, we now develop a new mechanism for branched HCR that enables label-free multiplexed sensing. The method is based upon increasing the dimensionality of the linear HCR systems by replacing the linear constructs used in conventional HCR assays with branched DNA structures. To demonstrate the principle of this transition from a 1D chain amplification to one with an increased *structural dimensionality*, we use a three-arm branching DNA construct^11^ to create “hybridization tails” as sites to initiate a chain reaction in the primer sequence. Although other mechanisms allowing the formation of branched structures have been developed recently, to take advantage of the exponential growth kinetics^12^, they have relied on complex fluorescence labeling strategies^13,14^ for label-based biosensing^15^, which often requires stringent storage conditions, and have not been capable of being multiplexed. We show that our new DNA construct enhances the chain reaction efficiency, as the initiator nucleic acid strand is recycled (i.e., as the “target”) during the branching process (Fig. 1). We show that the reaction progresses efficiently in dilutions of whole blood, demonstrating the stability of reagents in biological fluids and providing a route toward application in biomedicine. We further demonstrate for the first time, that the resulting polymers can, in contrast to previously published linear and branched HCR mechanisms, be readily detected by exploiting a growth mechanism that leads to a highly branched construct and results in an increased intrinsic viscoelasticity of the construct^16^. This requires less stringent storage conditions and enables label-free acoustic mass sensing, that would not otherwise be possible for other linear HCRs. The sensing mechanism uses SAW on a disposable chip, generated by interdigitated transducers (IDT). These electrode structures have been used extensively in analytical applications^17,18^. We also demonstrate that this new branched mechanism allows us to design a multiplexed detection strategy that requires fewer DNA primers, thus decreasing the probability of cross talk, that has previously limited this capability when linear HCR has been used, for example.

## Materials and methods

### HCR reaction

Primers, from Eurofins Genomics (detailed sequence information in Table S1), were tested for hybrids and hairpin structures using the Integrated DNA Technologies design tools (http://eu.idtdna.com/pages/scitools). The HCR reaction contained 0.1 μM of each primer (NP1/NP2/NP3) and initiator, 50 mM of Na_2_HPO_4_, and 0.5 M of NaCl. All the primers were heated upto 95 °C for 5 min and cooled down to room temperature naturally^6^, before adding the initiator. The HCR reaction was performed at room temperature overnight. The products of the reaction were analyzed in 3% agarose gels with SYBR gold (Invitrogen) in 1 × TBE buffer (Invitrogen). Gels were run at 75 V for 120 min and visualized under with SYGENE PXi. Anonymized EDTA Blood was obtained commercially (Cambridge Bioscience Ltd) from healthy volunteers and stored at 4 °C, until used and discarded after 8 days in storage.

### Fabrication of an acoustic biosensor

A SAW Love Wave (LW) biosensor, based on previously published designs^19^, was fabricated on the piezoelectric substrate (64° Y-X LiNbO_3_) by using standard photolithographic processes. A central sensing region (5 × 2 mm) between two inter-digital transducers (IDTs, 100 finger pairs, fabricated by liftoff of Au 100 nm/Ti 10 nm) was coated with 20 nm of gold, on top of a guiding PMMA layer (thickness 1156 nm) (Fig. S1). The working frequencies were in the range of 90–105 MHz. The biosensor response was characterized using an E5071C ENA series network analyzer (9 kHz–3 GHz), Agilent Technologies.

### DNA immobilization on Love wave sensor

A 5′-thiolated single-strand DNA (ssDNA) probe (D1), Table S1 in ESI, was bound to the gold sensing region (see Fig. S2 for a schematic view). The 25-nt (nucleotide) probe was fully complementary with the 3′ end of one common primer of the HCR (NP21). The ssDNA (10 μM) was first incubated with 100 mM Bond Breaker^TM^ TCEP solution (PIERCE) at room temperature for 2 h. The gold sensing areas of the biosensor were plasma-cleaned with oxygen (100 W, 20 s) before a 10-μL drop of ssDNA in 0.1 M potassium phosphate buffer (pH 3.8) was deposited onto it and incubated at room temperature for 5 h, leading to probe immobilization^20^. The remaining liquid was removed, and the surface washed exhaustively with the same buffer and blow-dried with N_2_. To prevent nonspecific binding the surface, the sensors were blocked with 1.0 mM aqueous solution of 6-mercaptor-1-hexanol (MCH), Sigma^21^, for 1 h. Finally, 6 μL of the HCR product was added for 30 min before again washing with buffer and drying with N_2_. Resonance frequencies were recorded before and after the addition of the HCR product. A positive reaction (initiator present), will result in both the NP2 DNA monomer and the HCR product 6 (Fig. 1) binding to the ssDNA probe, while for a negative reaction, only the NP21 monomer can bind. This causes a difference in the mass loaded onto the sensing region, which can be readily detected.

## Results

In one simple implementation, we are able to show how the branched polymer products, formed in situ on the surface of SAW–LW sensors^22^, can result in a simplified detection, when compared with conventional fluorescently labeled optical assays. We demonstrate the detection of a model target sequence (as the initiator) in whole blood and confirm a capability for multiplexing using gel electrophoresis.

Multiplexing HCR reactions requires that multiple target initiators give rise to independent specified amplified constructs in a single reaction without cross talk (where one initiator either amplifies or inhibits the other initiator’s constructs). We designed primers that create additional hairpin structures, based upon the catalytic formation of three-arm junctions^11^, which cross-react specifically upon initiation, yielding branching. This scheme opens up new applications for multiplexed DNA amplification, where a single reaction is able to detect the presence of a number of different initiators (or targets).

The key to achieving this is that the initiator structures were modified by the addition of a hybridization tail (sequences 1 and 1*) to two of the three metastable hairpin primers (NP1 and NP3) that enable junction formation and the production of a large branched polymer. Each primer, NP1, NP2, and NP3 has a stem of 18 base pairs enclosing a 12-nt loop, along with extra 6- and 20-nt-long sticky ends at their 3′ and 5′ ends, respectively. The 5′ 20-nt sticky end of NP1 (noted ‘1’ in Fig. 1) is complementary to the 5′ end of NP3 (‘1*’). In the absence of the 24-nt initiator, the monomers form stable hairpin structures that dominate the equilibrium kinetically, forming product 1 through hybridization of NP1 and NP3 together via the sequences 1 and 1*.

When the initiator, I0, was introduced into the assay, it activated a cascade of assembly steps with NP1, NP2, and NP3 undergoing a serial strand displacement reaction, where the hairpin structures were opened up to form three-arm branched junctions of Product 4 (Fig. 1) in a similar way as Yin et al.^11^. However, importantly, and in contrast to previously published HCR mechanisms, the formation of these structures included a strand displacement step that released the initiator. As a consequence, the free initiator again became available to catalyze further branching so increasing the efficiency of the reaction. Each three-arm branched product grew through the hybridization of the 5′ sticky ends of NP1 with that of NP3 (product 5), to produce a large polymer network (Product 6). The specific sizes expected for the products are summarized in Table S1. The process was kinetically driven toward a thermodynamic equilibrium through hybridization and was simply carried out by melting all DNA at ~95 °C for ~5 min, then cooling to incubation at room temperature (with no temperature cycling or thermal control necessary). Apart from the addition of the initiator, the reaction is “reagent-less” and does not require any sample manipulation.

**Fig. 2:**
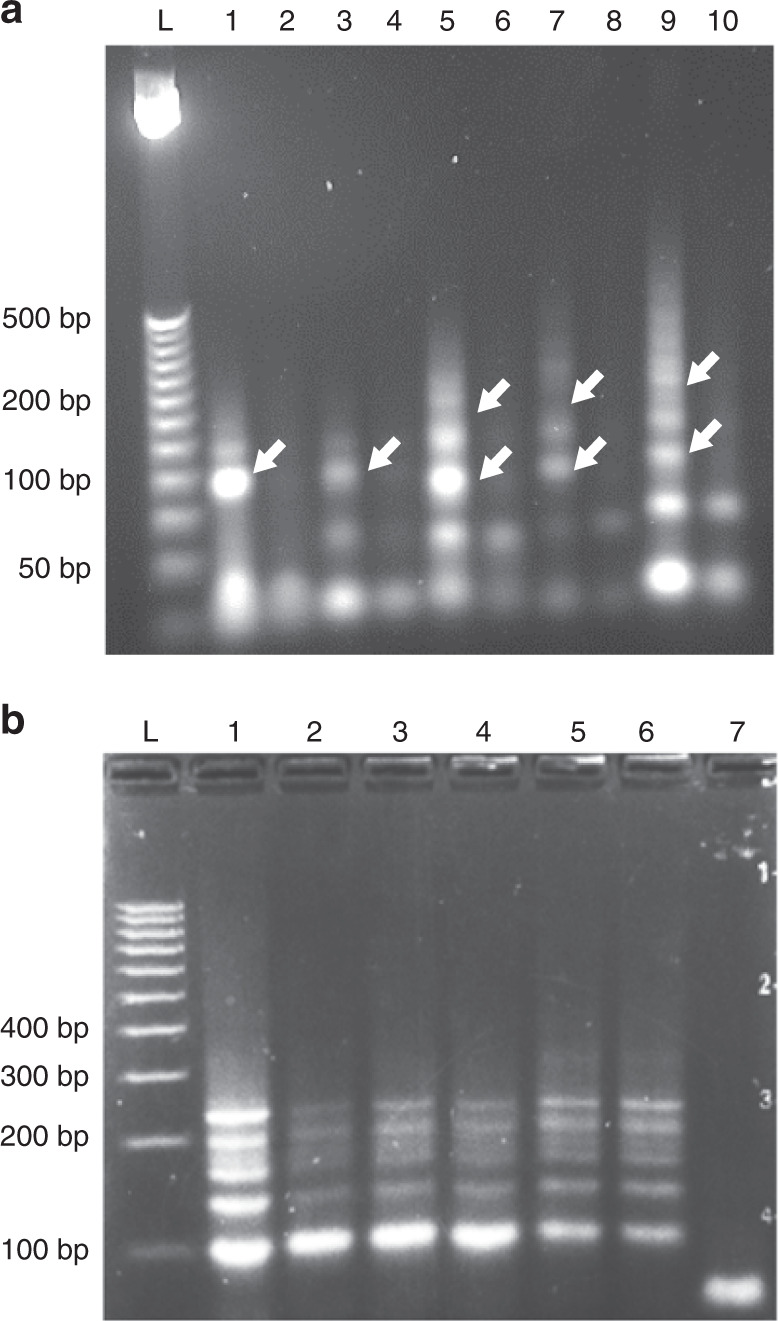
Gel electrophoresis of the branched HCR product. **a** HCR product with different tail lengths from 0 to 20 nt: lane L. 25-bp DNA ladder; lanes 2, 4, 6, 8, and 10 are negative controls without the initiator, while lanes 1, 3, 5, 7, and 9 are reactions with 1 µM of initiator; lanes 1 and 2–0-nt tail length; 3 and 4–5 nt; 5 and 6–10 nt; 7 and 8–15 nt; 9 and 10–20 nt. **b** HCR products obtained from reactions containing different volumes of human whole blood, spiked into 20-μL reaction volumes. Lane: 1–0 μL; 2–1 μL; 3–2 μL; 4–3 μL; 5–4 μL; 6–5 μL; 7—the negative control without any initiator. Lane L—100-bp ladder

Amplification by the proposed mechanism was dependent upon the binding between primers NP1 and NP3, which enabled the extension of the branches. Figure 2a shows that by increasing the length of each primer (from 5 to 20 nucleotides), this interaction can be strengthened, resulting in an increased efficiency of the reaction, as measured by the quantity of the largest branched product created. The negative controls of Fig. 2a (lanes 2, 4, 6, 8, and 10) show that when no initiator is present, only short-hairpin dimers can be formed (product 1 of Fig. 1), with minimal unspecific amplification when incubation time is increased significantly (e.g., 48 h). Since Lane 10 covers the reaction with the longest tails, product 1 is more readily visible than in other lanes that have shorter tails, at 74 bp. Similarly, Lane 1 also shows that when preventing the interaction between NP1 and NP3 (i.e., when there is no overlap), the branching does not occur, and the reaction stops at product 4 (81 bp)—similar to three-arm junctions^11^. Further increase of the tail lengths of the primers led to an increase in the size of the final product, as well as an increase in purity. For example, in Lane 9, the band relating to product 4 (101 nt) was hardly visible, while a smear of larger constructs can be seen (Fig. 2a—lane 9).

To validate the potential of this new assay in diagnostic applications, we showed that the branched HCR reaction proceeded when a target sequence (the initiator) in a 20-μl HCR reaction was added to finger prick the volume of blood. Figure 2b clearly illustrates that such small volumes of blood do not inhibit the reaction, paving the way to low-volume (finger-prick) biosensing.

**Fig. 3:**
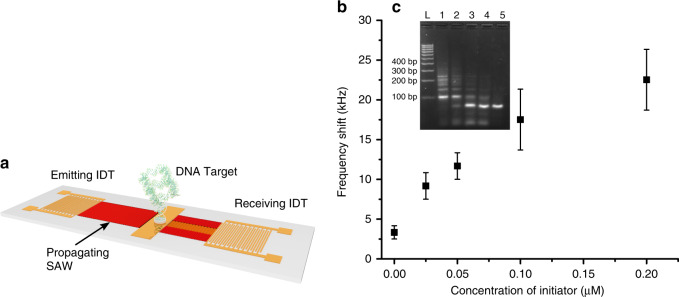
Label-free acoustic detection of the HCR product. **a** Schematic drawing of the SAW sensor, showing the opposing pair of the interdigitated transducer and the central gold sensing layer. **b** The frequency shift of the resonance frequency of the IDT pair, showing the effect of mass loading by the branched HCR product formed in situ on the sensor surface, for different initiator concentrations, showing a sensitivity down to 25 nM. Data are the average of at least three independent experiments and error bars represent the standard deviation. The limit of detection was defined as the first concentration above the background, with a confidence of 99% (3 standard deviations). **c** Initiator concentration dependence of the HCR product branching shown by agarose gel electrophoresis: L. 100-bp ladder; 1. 0.2 μM initiator; 2. 0.1 μM; 3. 0.05 μM; 4. 0.025 μM; 5. Negative (0 μM, ddH_2_O). Note that the ladder is the same as in Fig. 2b, but that the spacing between the bands differs

In its conventional implementation, linear HCR has been enabled using simple optical detection of DNA intercalating dyes^9^ or through more sensitive FRET detection using conjugated primers^23^. This new three-arm branched method uniquely enables the formation of higher- dimensional molecular networks of DNA, which provides the opportunity to implement this reaction onto a label-free sensor. We demonstrate this using an acoustic LW sensing strategy based upon generating SAW on a disposable chip and IDT. Here, we show the detection of the reaction product with monomer NP21 (Fig. 3), with a mechanism that is based on DNA hybridization between a ssDNA (capture probe, D1) immobilized on the surface of the biosensor, is able to recognize one of the primers used in the HCR reaction (Fig. S2). The 3′ sticky end of NP21 is complementary to the capture probe, enabling the branched HCR to attach to the surface of the biosensor via a sandwiched hybridization.

Without any reaction (no initiator), only the small primer NP21 can bind to the surface, yielding a small frequency shift (3.3 kHz—Fig. 3b, 0 µM). In contrast, when an amplification reaction occurs by addition of an initiator, the large nucleotide copolymers “grow” on the chip surface, changing the mass loading of the sensor. This molecular bulk resulting from the increased dimensionality within the construct leads to a frequency shift to lower-resonance frequencies (Fig. 3b). We demonstrated the sensing of the target initiator through the detection of the amplification reaction product at a concentration down to 25 nM (Fig. 3b), as a similar order of magnitude to previously published work^12^. Further, Fig. 3b shows that the signal increases with increasing initiator concentration. Our HCR system is kinetically rate limited and by increasing the initiator concentration (e.g., above 200 nM in Fig. 3c), the reaction is more rapid, yielding more constructs and increasing the extent of the branching (see Fig. 3c confirming that at higher initiator concentrations, the amount of product increases).

**Fig. 4:**
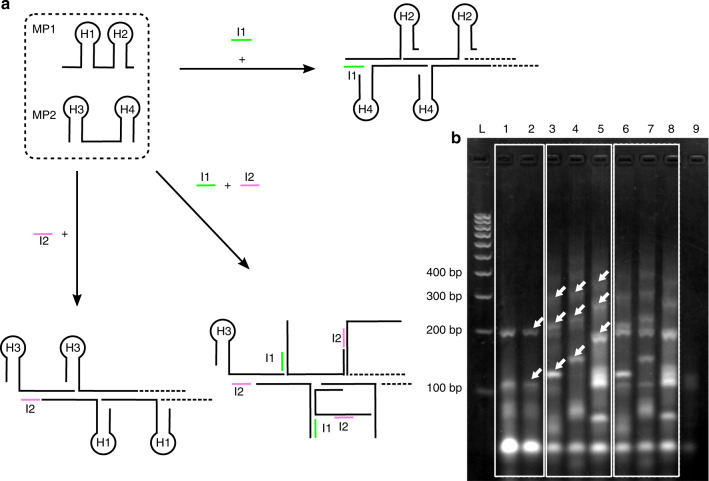
Multiplexed HCR mechanism. **a** Primers MP1 and MP2 each contain two hairpin structures H1–2 and H3–4, respectively. Upon adding initiator I1, loops H1 and H3 promote a linear HCR amplification. Similarly, initiator I2 interacts with H4 and H2. When both initiators are present, the reaction results in a highly branched structure (Fig. S3). **b** Agarose gel electrophoresis of the multiplexed reaction. The lanes are as follows L: 100-bp ladder; 1–8 HCR reactions with different initiators (all concentrations are 0.1 µM). Lane 1. Only initiator I1 added; 2. Only initiator I2; 3–5. Initiator I2 of increasing size (25, 49, and 118 nt); 6–8 both initiators are added, with I2 of increasing size for each lane (25, 49, and 118 nt). Lane 9 negative control (no initiator). Arrows indicate the first band of the first two products for each experiment. Note that as the size of the initiator is increased, so does that of the product

As already stated, multiplexing linear HCR reactions, where multiple initiators give rise to independently specified amplified constructs in a single reaction, require a complete set of hairpin primers for each initiator to be detected^8,24^. This design challenge leads to high probabilities of cross talk, where one initiator either amplifies or inhibits the other’s constructs (leading to unspecific amplification or false-positive reactions). Here, using the branched HCR, we show that by increasing the dimensionality of the amplification product, we were able to design a new class of a multiplexed HCR mechanism, which requires only two primers (MP1 and MP2) to detect two different initiators (I1 and I2—Fig. 4a, and in more details in Fig. S3). In a linear HCR, such an assay would require four different primers. The addition of a I1 binds to primer MP1 and opens the loop H1, which is then available to open loop H3 of primer MP2 in a conventional HCR mechanism. Conversely, I2 is able to open loop H4 of primer MP2, which triggers amplification via the opening of loop H2 of MP1. When both initiators are present in the reaction, all loops are opened and a complex branched structure is created. The details of the sequences and binding reactions are provided in Fig. S3 and Table S1.

We confirm the multiplexing capability of this design using gel electrophoresis, Fig. 4b. To demonstrate that our multiplex HCR reaction was able to distinguish two different targets, initiator 2 (I2) with different sizes was prepared. Lane 9 provides the results from a negative control, with no initiator present, showing small amounts of nonspecific primer–dimer complexes. These were also present in the other lanes (1–8), where initiators were added to the reaction. In lane 1, only initiator 1 was added and only initiator 2 in lane 2. These lanes show bands characteristic of the first stage of the mechanism at 108 bp, as well as bands of the further stages (e.g., at 204 bp).

To investigate the mechanism of this cascade in detail, we varied the length of initiator 2 at the 5′ end, accordingly: 25 nt (lane 3), 49 nt (lane 4), and 118 nt (lane 5). The results show an increase in the size of the DNA structures created through the reaction, in line with the proposed mechanism (Fig. S3), directly following the increase in initiator length and showing the intermediate stages within the extension of the branched DNA (as indicated by white arrows in Fig. 4b). Lanes 6–8 show the results of the reaction with both initiators (I1 and I2) present, for increasing sizes of I2, demonstrating a successfully multiplexed amplification. Here, the band structure is more complex, reflecting the increased dimensionality of the amplicons, but still shows bands linked to structures proposed in the mechanism (Fig. S3). Multiplexing to higher orders (three targets and more) requires the addition of a primer and the elongation of the MP-type sequences to account for the additional binding (above 120 nt), the design of which would need particular care to avoid unwanted and strong secondary structures. This multiplexing strategy is analogous to an OR logic gate, detecting if any of the inputs are present. Analytically, such a device would have potential applications in triage strategies for example, where patients can be oriented toward more specific (and expensive) diagnostic tests following a low-cost initial screen.

## Conclusion

In conclusion, we have demonstrated a new high dimensionality or branched HCR reaction that, unlike PCR or isothermal amplifications, neither requires enzymes nor precise temperature control. The presence of an initiator, which may be an analyte of interest, initiates a cascade reaction, to produce a network of interlinked DNA products. In order to demonstrate that this offers a route for enzyme-free, label-free DNA detection, we detected a model target sequence (as the initiator) added to a finger-prick of whole blood. Our technique could provide a significant advance in the detection of circulating tumor DNA for example, an application already amenable to HCR^25^, but which would greatly benefit from multiplexing. We also demonstrated a capability for branched HCR to enable multiplexed detection, using a single set of two primers to negate the off-target effects and false-positive amplifications that currently limit the application of conventional linear HCR technologies.

## Supplementary information

Supplementary Data
