# Supplementary material for: Branched hybridization chain reaction—using highly dimensional DNA nanostructures for label-free, reagent-less, multiplexed molecular diagnostics
Source: Microsyst Nanoeng. 2019 Aug 12;5:37. doi: 10.1038/s41378-019-0076-z (PMC6799823; doi:10.1038/s41378-019-0076-z)
Supplement: Supplementary file 1 — Supplementary Data [file 41378_2019_76_MOESM1_ESM.docx]

Supplementary Information

Branched Hybridization Chain Reaction – using highly dimensional DNA nanostructures for label-free, reagent-less, multiplexed molecular diagnostics.

Gaolian Xu, Mingliang Lai^±^, Rab Wilson, Andrew Glidle, Julien Reboud and Jonathan M. Cooper^*^.

**Supplementary data.**

**Table S1.** Product sizes for singleplex branched HCR. The product number is referred to in Figure 1.

| Product number | Product size (in bp) |
| --- | --- |
| 1 | 101 |
| 2 | 202 |
| 3 | 303 |
| 4 | 404 |
| 5 | 505 |


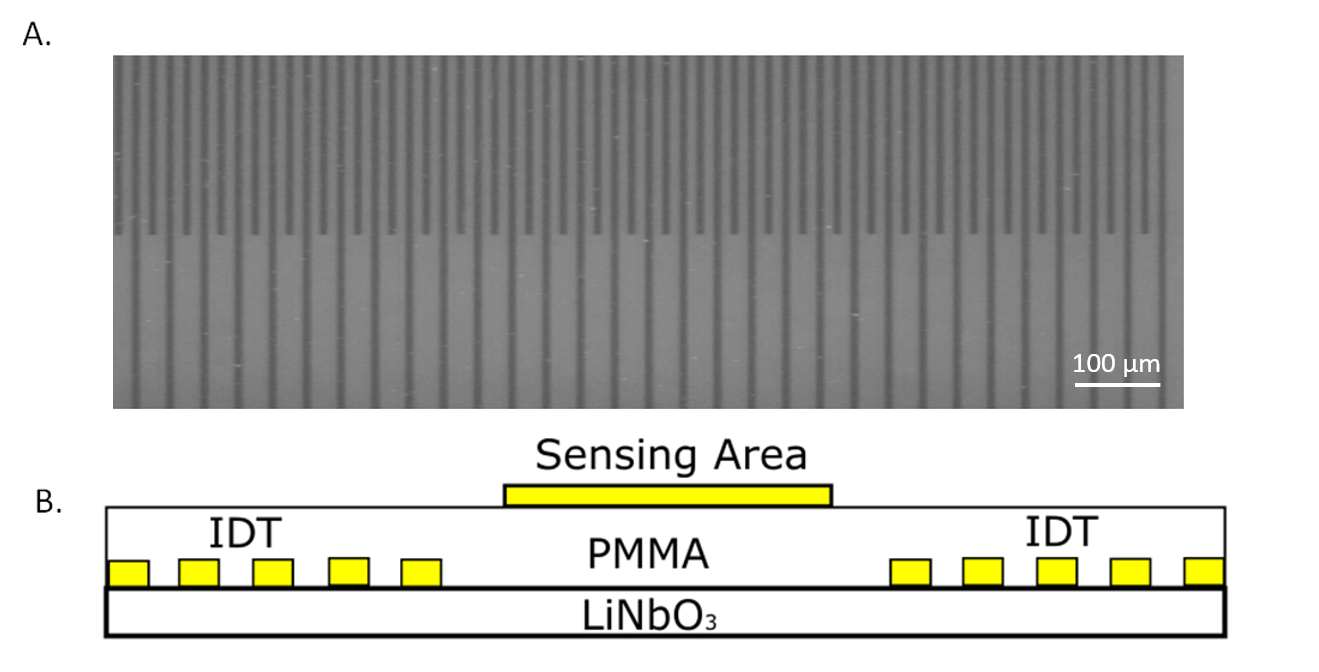


**Fig S1.** Love Wave (LW) biosensor. (A). Picture of part of the interdigital transducer (IDT) of the LW biosensor, the scale bar is 100 µm (frequency ca. 100MHz); (B). Scheme showing the cross Section of the LW biosensor with a PMMA guiding layer and sensing area covered with gold thin film.


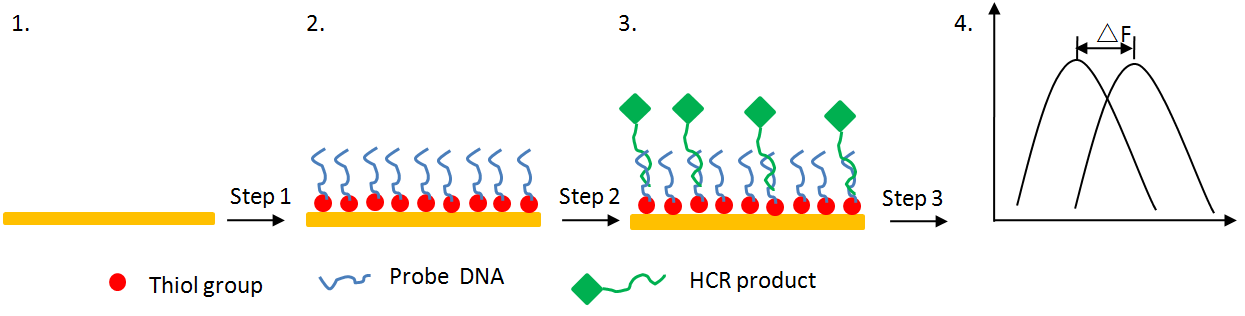


**Fig S2.** Schematic diagram of HCR product detection using a LW biosensor: 1. The gold sensing area was cleaned with oxygen plasma; 2. Immobilization of the single stranded DNA probe on the surface of the sensing area through thiol chemistry; 3. Hybridization between immobilized probe and HCR product amplified in the suspension above the sensor; 4. The measurement involves comparing the resonant frequency before and after the addition of the HCR product, providing a frequency shift (∆F) which is proportional to the amount of bound HCR product.

**Fig S3** (next page). Mechanism of multiplex HCR reaction. (A).The design of monomers and initiators for multiplex HCR. Fragments labelled with a number and a ‘*’ are complementary to the ones with the same number but no ‘*’ (e.g. fragment 2 of MP1 is complementary to 2* on MP1, forming a hairpin structure). Each primer (MP1 and MP2) has two hairpin structures which coexist until the introduction of different initiators (I1 and I2); The 5’ end of MP1 has a stick sequence (1) which is complementary to I1, and the middle of MP2 has a sequence (5*) complementary to I2. The hybridization between I1 and MP1 (or I2 and MP2) opens the hairpin structures and starts serial catalytic reactions to form products with different structures. Detailed information is list in Table SII (B). Structures formed when I1 only is introduced in the reaction. (C). Structures formed when I1 only is introduced in the reaction. (D). Structures formed when both I1 and I2 are introduced in the reaction. With the introduction of the initiator (I1 for example), the 3’ end of I1 (1*) complementary to the 5’ sticky end of MP1 (1) leads to an unbiased strand-displacement interaction to open the first hairpin structure of MP1 but maintains the second hairpin structure. The strand displacement process will expose the fragment of 3 and 2* in MP1. The part 3 and 2* in MP1 can hybridize with the 3* of MP2 and starts another strands displacement process, exposing the 1* and 2* in the MP2. The exposed 1* and 2* will be then served as an initiator to start another round of strand displacement reactions, creating a branched structure.


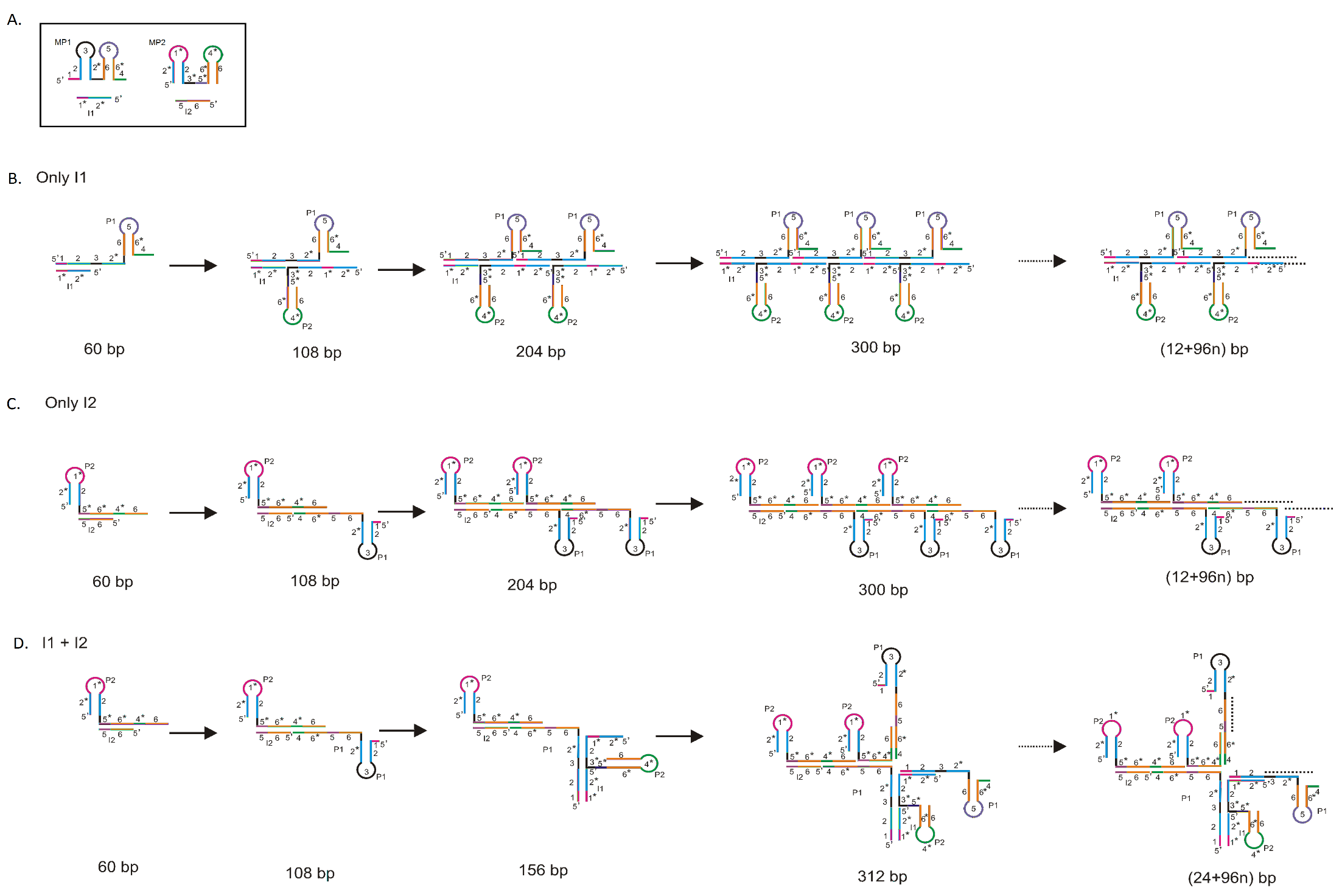


**Table S2.** Novel HCR and Multiplex HCR primers sequence information

| System | Name | Sequence (5'-3') |
| --- | --- | --- |
| Novel HCR |  |  |
|  | NP1 | 5-CGGCGG-CAGGGT-TGATAG-TCATAG-TCCAAT-CACAAC-CTATGA-CTATCA-ACCCTG-AACCACCACCAACCACCACC |
|  | NP2 | 5-TGATAG-TCATAG-GTTGTG-ATTGGA-ACCCTG-CCGCCG-TCCAAT-CACAAC-CTATGA |
|  | NP3 | 5-GTTGTG-ATTGGA-CGGCGG-CAGGGT-CTATGA-CTATCA-ACCCTG-CCGCCG-TCCAAT-GGTGGTGGTTGGTGGTGGTT |
|  | NP21 | 5-TGATAG-TCATAG-GTTGTG-ATTGGA-ACCCTG-CCGCCG-TCCAAT-CACAAC-CTATGA-TCTGAGGATAAGCGCTCCGGTG |
|  | D1 | 5'-SH-TATGCACCGGAGCGCTTATCCTCAGA |
|  | I0 | 5-CTATGACTATCAACCCTGCCGCCG |
| Multiplex HCR | MP1 | 5-TTAACCCACGCCGAATCCTAGACTCAAAGTAGTCTAGGATTCGGCGTG-T-GGAACAGCTTTGAGGTGCCATCTCGCACCTCAAAGCTGTTCCACTGTG |
|  | MP2 | 5-AGTCTAGGATTCGGCGTGGGTTAACACGCCGAATCCTAGACTACTTTG-T-GAGATGGCACCTCAAAGCTGTTCCCACAGTGGAACAGCTTTGAGGTGC |
|  | I1 | 5-AGTCTAGGATTCGGCGTGGGTTAA |
|  | I2  I2-1  I2-2  I2-3  I2-4 | 5-GGAACAGCTTTGAGGTGCCATCTC  5-TGCACCGGAGCTTTGCTCCGGTGCAGGAACAGCTTTGAGGTGCCATCTC  5-TCTGAGGATAAGCGCTCCGGTGCATAGCTTTGCTATGCACCGGAGCGCTTATCCTCAGAGGAACAGCTTTGAGGTGCCATCTC  5-CCACCAGCTAGATGTTGAAGGACTGGACGAGCTGATTACGACTCAGCGACAGATTTGTGGGAACAGCTTTGAGGTGCCATCTC  5-CACAAATCTGTCGCTGAGTCGTAATCAGCTCGTCCAGTCCTTCAACATCTAGCTGGTGG |

Note: The sequences (NP1, NP2, NP3 and NP21) are also listed as text sequences annotated with segment names in the text. The underlined fragments are the sticky end for hybridization between the three-arm branched products.
